# Pathologic complete response to TNT + camrelizumab for rectal cancer with surgical anus-preservation: case report and literature review

**DOI:** 10.3389/fsurg.2023.1192569

**Published:** 2023-07-04

**Authors:** Feng Pi, Gang Tang, Chaozheng Xie, Yukun Cao, Shilai Yang, Zhengqiang Wei

**Affiliations:** Department of Gastrointestinal Surgery, The First Affiliated Hospital of Chongqing Medical University, Chongqing, China

**Keywords:** anus-preserving, camrelizumab, chemoradiotherapy, locally advanced rectal cancer, total neoadjuvant therapy

## Abstract

**Background:**

This case report demonstrates the efficacy of total neoadjuvant therapy (TNT) based on pathological complete response (PCR). We also discuss the surgical approach to preserving the anus and its perioperative management.

**Case presentaion:**

The patient was a 26-year-old woman, with blood in the stool and stool thinning for over two months. Preoperative examination revealed locally advanced rectal cancer invading the left anal raphe and enlarged lymph nodes adjacent to the left internal iliac vessels. The lesion was preoperatively classified as T4bN1bM0 IIIC. Considering the size and depth of the tumor, it was difficult to have sufficient margins for radical resection, and the tumor was too close to the anal orifice. Considering the patient's youth and strong desire to preserve the anus, it was decided to use TNT combined with a camrelizumab regimen. After the entire course of neoadjuvant radiotherapy, the tumor size significantly reduced in fibrotic manifestations, and no enlargement of the lymph nodes adjacent to the left internal iliac vessels was observed. She underwent robotic laparoscopic ultra-low anterior rectal resection, left lateral lymph node dissection, and temporary ileostomy, and no significant residue was observed after all bowel tubes were taken for examination, nor was there cancerous involvement at the distal or radial cut edges, or metastasis. The patient was discharged nine days postoperatively, and no major complications were detected. Follow-up was performed without adjuvant chemotherapy.

**Conclusions:**

TNT may be a better surgical option for preserving the anus and for complete radical resection in patients with LARC for whom Miles’ resection is indicated.

## 1. Introduction

Rectal cancer is a common disease worldwide, accounting for one-third of all colorectal cancers (1). Local recurrence after rectal cancer surgery has historically exceeded 20% at 2 years, with locally invasive lesions harboring the highest risk (2). Surgery is the cornerstone of rectal cancer treatment, and total mesorectal excision (TME) is the gold standard (3, 4). However, for locally advanced rectal cancer (LARC), it is difficult to accomplish the purpose of radical surgery using surgical excision alone, which has become a challenge for surgeons (4, 5). Increasing the role of neoadjuvant therapy rather than postoperative adjuvant chemotherapy in rectal cancer can achieve better tolerance and pathological complete remission (PCR) (6), a concept known as total neoadjuvant therapy (TNT) (7). Recently, immunotherapy has been shown to play an important role in cancer treatment. In particular, PD-L1 inhibitors (e.g., camrelizumab) and PD-1 antibodies can block malignant tumor cells, tumor-infiltrating lymphocytes, and tumor-infiltrating dendritic cells by targeting PD-1 on the surface of immune cells (8–10). They have shown promising efficacy in treating rectal cancers with high levels of microsatellite instability (MSI-H) (11). Although immunization alone is less effective than desired for microsatellite-stable (MSS) rectal cancers(12, 13), combining PD- L1 inhibitors (e.g., camrelizumab) with other radiotherapy regimens has shown impressive results in some advanced MSS rectal cancers (14, 15). Therefore, the significance and efficacy of preoperative chemoradiotherapy strategies for LARC, especially microsatellite-stable cancer (MSS), remain controversial. To date, there are no reports showing the efficacy of camrelizumab in combination with TNT for LARC with microsatellite stability.

In this case report, we demonstrate the apparent effectiveness of TNT + immunotherapy and the advantages of organ preservation through TNT, including a short course of radiotherapy + XELOX + camrelizumab, and radical surgery, as shown by pCR and anus preservation. In addition, we review the relevant literature and cases to discuss the surgical approach (with anus preservation) and perioperative management.

## 2. Case presentation

**Figure 1:**
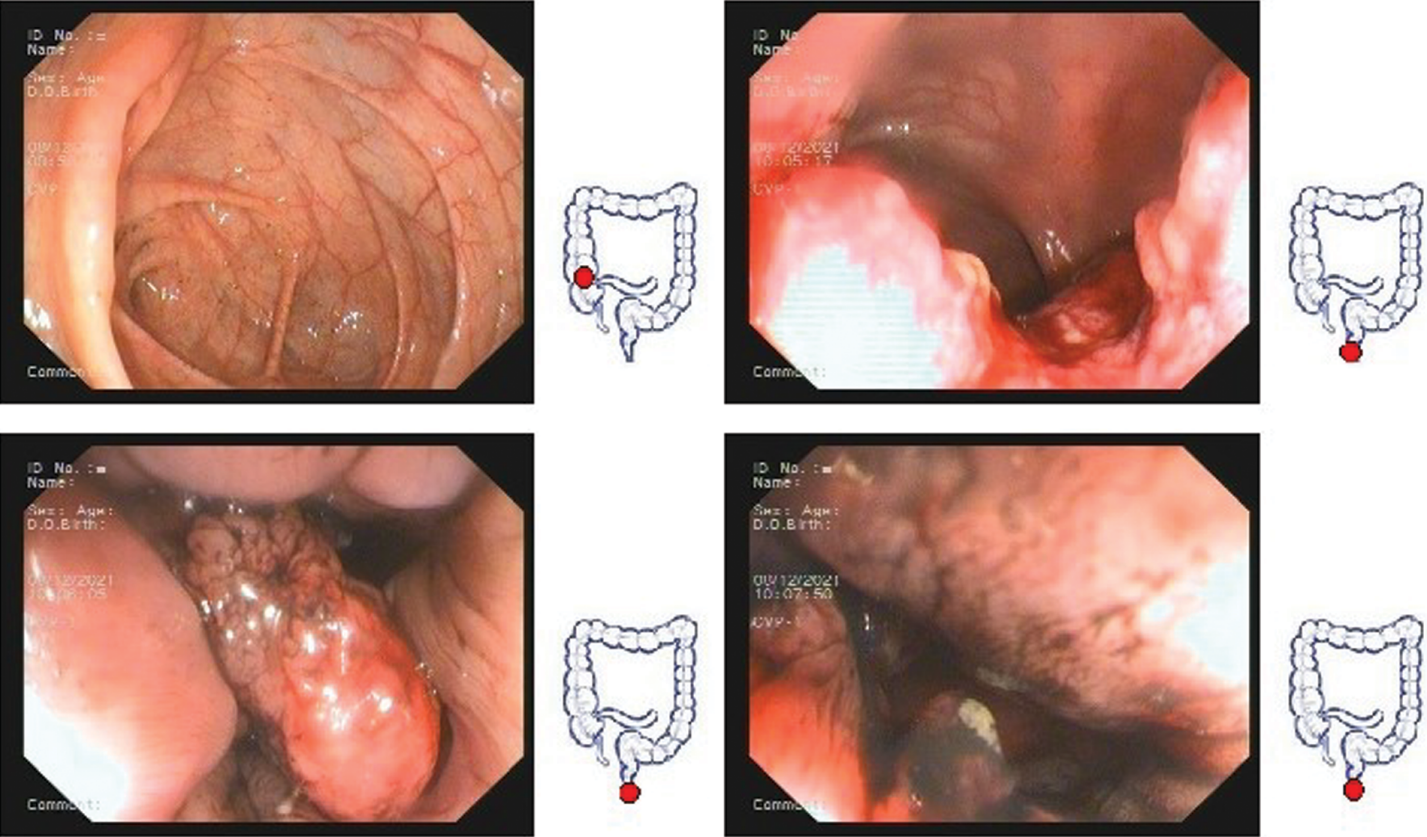
Colonoscopy images before neoadjuvant therapy.

**Figure 2:**
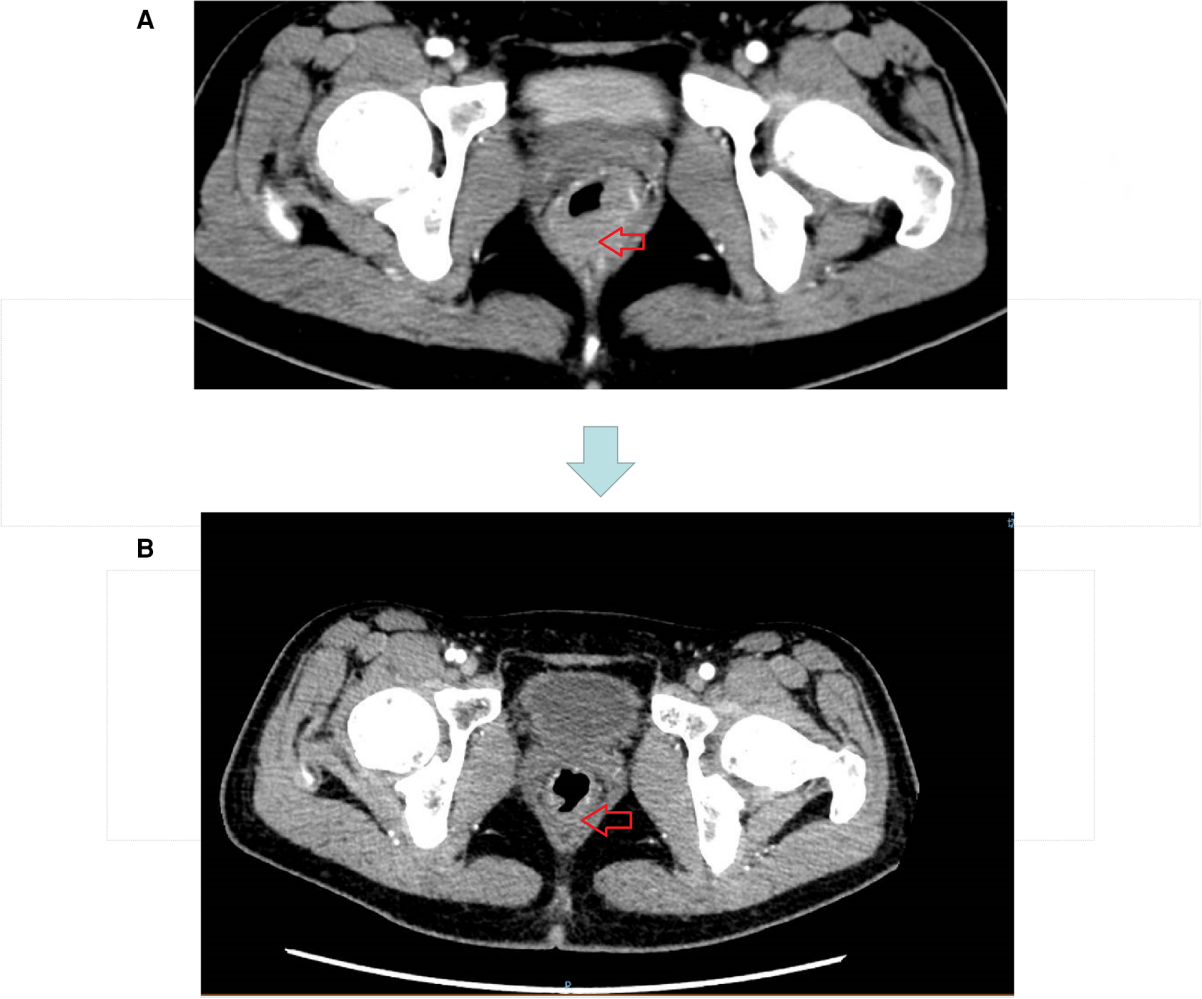
**(A)** CT image before neoadjuvant therapy. **(B)** CT image after three cycles of short course radiotherapy + XELOX + camrelizumab.

**Figure 3:**
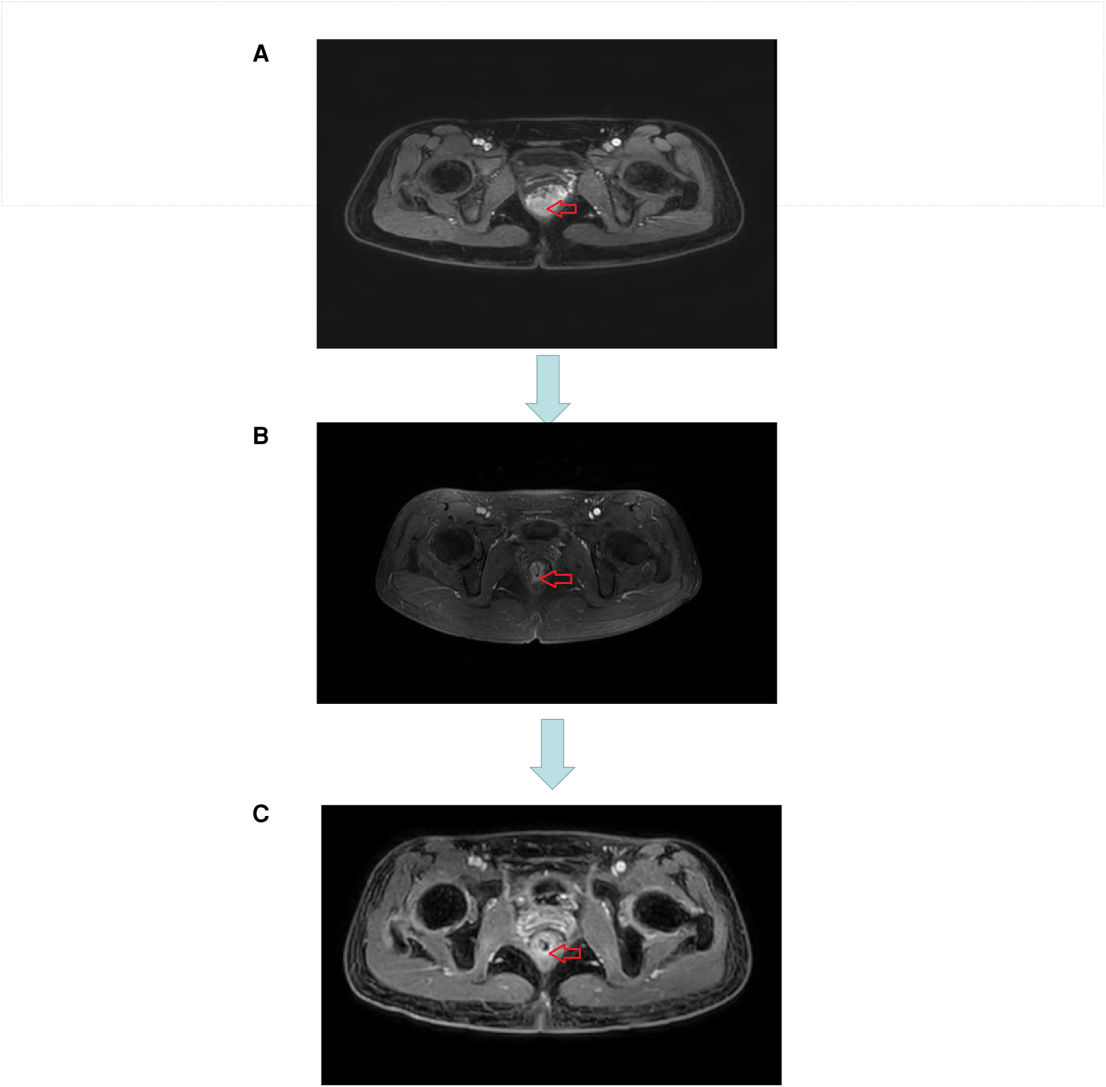
**(A)** MRI image before neoadjuvant therapy. **(B)** MRI image after three cycles of short course radiotherapy + XELOX + camrelizumab. **(C)** MRI image at the end of neoadjuvant therapy.

Our patient was a 26-year-old woman with blood in the stool and thinning for 2 months, with a feeling of urgency and heaviness. No family history of cancer. Laboratory findings revealed a hemoglobin concentration of 101 g/L. Serum carcinoembryonic antigen and carbohydrate antigen 19–9 levels were 2.2 ng/ml and 7.8 U/ml, respectively. Colonoscopy revealed a cauliflower-like neoplasm in the lower part of the intestine, with necrosis and hemorrhage on the surface, hard and poorly mobile, and the lower margin adjacent to the anal dentate line, invading most of the perimeter of the intestinal lumen (Figure 1). Magnetic resonance imaging (MRI) showed: The lower margin of the tumor was about 1.9 cm from the anal margin, the tumor invaded the left anal levator muscle, and the left internal iliac artery lymph node was enlarged; CRM (circumferential resection) (+), EMVI (extra-mural vascular invasion) (-) (Figure 3). No distant organ metastasis was observed on computed tomography (CT) (Figure 2), and the lesion was preoperatively classified as stage T4bN1bM0 IIIC according to the Chinese CSCO guidelines for colorectal cancer (16).

**Figure 4:**
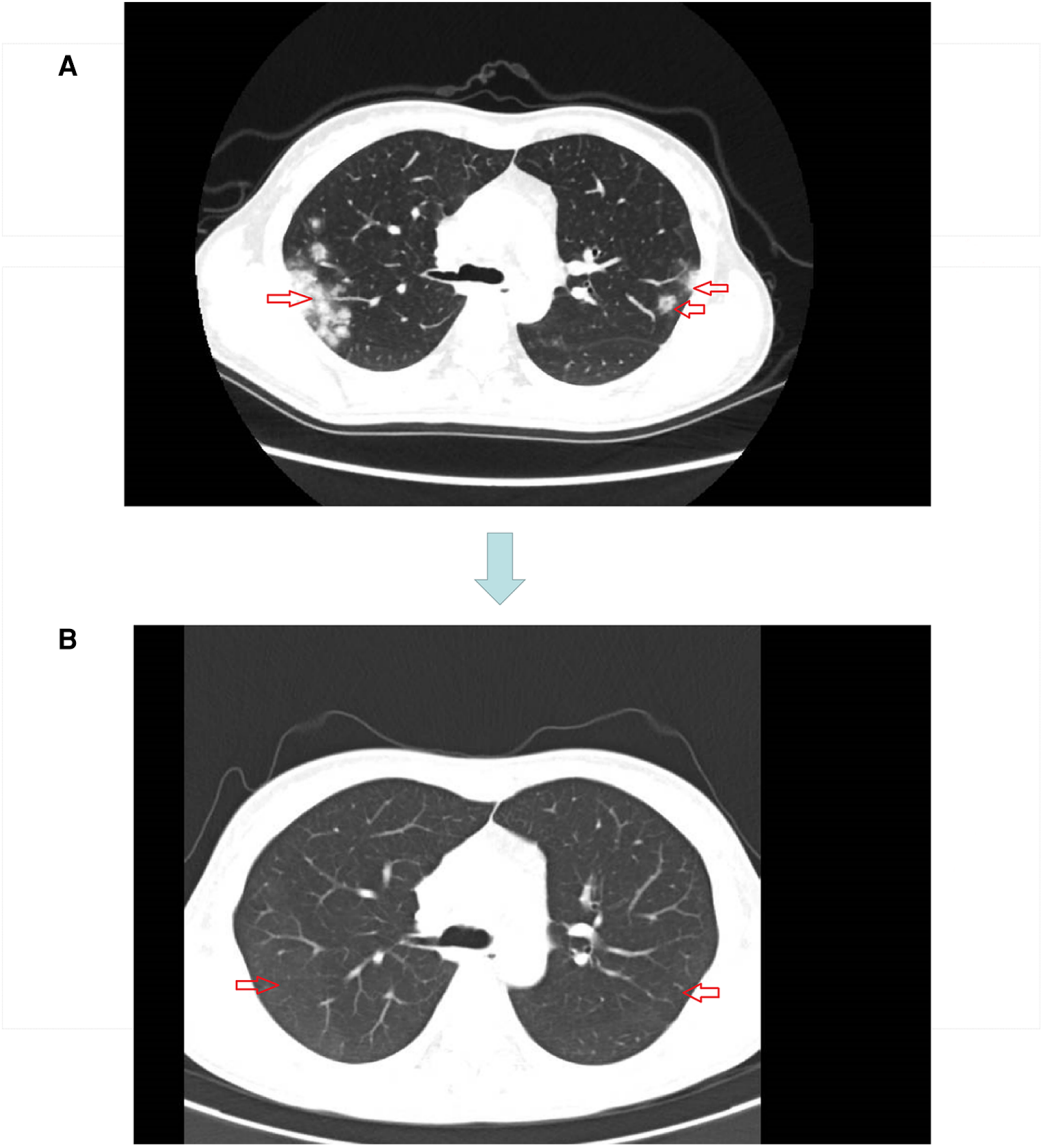
**(A)** Lung image after three cycles of short course radiotherapy + XELOX + camrelizumab. **(B)** Lung image one month after discontinuation of camrelizumab.

The original treatment plan we considered was CRT in anticipation of tumor shrinkage, followed by radical rectal cancer surgery (Miles) (17, 18), but considering that the patient was young and had a strong desire to preserve the anus, we decided to perform total neoadjuvant therapy (TNT) after multidisciplinary team discussions. This is not a routine treatment for high risk LARC in our hospital, however, we expect to make the preoperative treatment more effective with immunotherapy + TNT, we used a short course of high-dose radiotherapy plus the immunotherapy drug camrelizumab, as follows: a short course of radiotherapy (5 Gy, 5 days) followed by XELOX ((capecitabine 1500 mg/m^2^ bid for 14 days and L-OHP 130 mg/m^2^ on day 1))+ camrelizumab was administered every 3 weeks for 3 cycles. Review after three cycles suggested that the lesions were significantly smaller and the left internal iliac vessel parietal lymph nodes were smaller than before the treatment (Figure 3). However, multiple patchy shadows were observed in both lungs (Figure 4). We concluded that this might be due to immune-related pneumonia caused by camrelizumab; therefore, camrelizumab was discontinued and XELOX alone was administered every three weeks for three cycles. It is worth mentioning that chest CT was reviewed one month after discontinuing camrelizumab. Most multiple patchy shadows on the lungs disappeared (Figure 4).

After completion of TNT, the review CT suggested that the original tumor was only seen as a few low-signal foci of fibrosis, MRI suggested that the stage was Tx N1a, CRM (-), EMVI (NA), and the primary tumor MR TRG grading (standard) was about grade 1 (grade 1: completely low-signal fibrotic component, no residual isosignal tumor tissue) (Figure 3), rectal finger examination is about 7 cm into the finger, no mass is found, half circle of tumor scar can be found at about 3 cm–4 cm from the anus. laparoscopic ultra-low rectal anterior resection + lateral lymph node dissection + temporary ileostomy was performed with robotic assistance. No intraperitoneal dissemination or distant metastasis was observed. Low anterior resection (LAR) was used to ensure adequate tumor margins by means of rectal finger examination with the assistance of an assistant during distal bowel dissection, and the specimen was sent for frozen biopsy and this suggested that there were no tumor cells in the distal rectal cut margin. The patient was discharged 9 days after the surgery without major complications or postoperative events. Pathological examination of the resected specimen revealed no significant residues in the intestinal canal. No cancerous involvement was observed at the distal or radial cut edges. No cancer metastasis was observed in the internal or lateral iliac lymph nodes (0/1/0/6), and no metastasis was observed in the mesorectal lymph nodes (0/13). Therefore, the rectal cancer was diagnosed using PCR, and the anus was successfully preserved. Three months later, she underwent ileostomy closure surgery and was discharged five days after surgery. The patient was followed up without adjuvant chemotherapy and survived without recurrence at the 9-month follow-up.

## 3. Discussion

We have described a rare case of a patient with surgically resected locally advanced rectal cancer who showed impressive PCR results after TNT, with preservation of the anus as a vital organ.

In the current treatment strategy for LARC, we are increasingly focusing on the role of TME and neoadjuvant therapy (19–21), particularly TNT, which has led to more benefits in these patients, including local tumor control and significant improvement in survival and even quality of life (22). In Japan, a multicenter phase II trial of NAC with XELOX plus bevacizumab for locally advanced rectal cancer reported satisfactory short-term outcomes.

A multicenter phase II trial of NAC with XELOX plus bevacizumab for LARC reported satisfactory short-term outcomes, with a completion rate of 84.4% and pCR rate of 13.3% (23). Cercek et al. demonstrated in NEJM that single-agent immunotherapy can achieve a Pathologic complete response to preoperative neoadjuvant therapy for certain colorectal tumors (24). A phase II trial of nivolumab in combination with preoperative radiotherapy similarly reported improved PCR rates with preoperative CRT, nivolumab, and radical surgery (25); therefore, we believe that stronger and longer preoperative neoadjuvant therapy (TNT + camrelizumab) may provide patients with higher survival rates and the opportunity to preserve vital organs.

Difficulties with TNT + camrelizumab in the treatment of locally advanced rectal cancer must be addressed. First, although immunotherapy improves outcomes in CRC patients, it also leads to an increase in unique immune-related adverse events (irAEs) (26, 27), as in this case, so camrelizumab-induced immune pneumonitis must be of concern to surgeons. Therefore, it is recommended that chest CT be performed once per cycle of chemotherapy, in the event of immune pneumonia, based on previous clinical experience, we recommend that patients with poor general condition not tolerating follow-up therapy or severe respiratory symptoms may choose to discontinue Camrelizumab, and if they have mild symptoms and general condition tolerating follow-up therapy, they may be treated with methylprednisolone and Mycophenolate mofetil (28), and then continue to complete TNT. We tend to view immunotherapy as an adjunct to TNT, and therefore discontinuation is acceptable if associated risks arise. In addition, attention should be paid to the destruction of ovarian hormones in female patients by radiotherapy in localized areas, which may result in symptoms such as menopause and infertility in young patients (29). Alternative regimens, such as pre-radiation ovarian suspension or egg freezing are recommended to prevent these conditions (30).

Second, there are difficulties associated with long-course radiotherapy and chemotherapy during surgery. During surgery, the anatomy of the patient's surgical area is confusing because of prolonged radiotherapy, chemotherapy, and traumatic bleeds, which greatly increase the difficulty of radical resection. Therefore, careful differentiation of anatomical structures to complete TME requires a surgeon with excellent clinical surgical skills. Third, anal function must recover after radical resection. One of the most important concerns of ultralow anal preservation surgery is the ability to continence (31, 32). Therefore, to prevent anal incompetence, we carefully separated the nerves and blood vessels during the surgery and created a colonic J-pouch (33, 34) to replace the original rectal ampulla. Eight months after radical surgery (5 months after ileal re-entry), the patient's ability to control stool was gradually restored, as shown by less frequent stools and gradually formed and controlled stools. Finally, patient tolerance to and compliance with a prolonged preoperative treatment regimen was assessed. We hope to bring the attention of our medical colleagues through case reports to benefit patients, especially young colorectal cancer patients. Of course, we will follow up with this patient further to assess the benefits and harms of this treatment option. Because this is a report of a single case, future clinical trials are warranted.

In conclusion, a patient with LARC was treated with TNT + camrelizumab and underwent PCR and complete radical resection with preservation of the anus. She remains alive 9 months after surgery without recurrence. TNT + camrelizumab may be a better surgical option for anal preservation and complete radical resection in patients with LARC who have indications for Miles' surgery.

## Data Availability

The original contributions presented in the study are included in the article, further inquiries can be directed to the corresponding author.
